# In-silico trials of targeted screening for abdominal aortic aneurysms using linked healthcare data: A study protocol

**DOI:** 10.1371/journal.pone.0327856

**Published:** 2025-07-16

**Authors:** Liam Musto, Athanasios Saratzis, Mintu Nath, Emmanuel Katsogridakis, Ann Elsworth, Sylwia Bujkiewicz, Clark Hobson, Claire Lawson, Susan Wallace, Maria Gonzalez-Aguado, Aiden Smith, Susan Hodgson, Guiqing Lily Yao, José Miola, Matthew J. Bown

**Affiliations:** 1 Department of Cardiovascular Sciences, NIHR Leicester Biomedical Research Centre and BHF Centre for Research Excellence, University of Leicester, Glenfield Hospital, Leicester, United Kingdom; 2 Biostatistics Research Group, Department of Health Sciences, University of Leicester, Leicester, United Kingdom; 3 School of Law, University of Leicester, Leicester, United Kingdom; 4 Department of Health Sciences, University of Leicester, Leicester, United Kingdom; 5 Medicines and Healthcare Products Regulatory Agency, London, United Kingdom; PLOS: Public Library of Science, UNITED KINGDOM OF GREAT BRITAIN AND NORTHERN IRELAND

## Abstract

**Background:**

The NHS abdominal aortic aneurysm (AAA) screening programme (NAAASP) is both clinically and economically effective. One of the main determinants of this effectiveness is disease prevalence. AAA prevalence is decreasing over time, steadily reducing the efficiency of the current NAAASP screening policy. One alternative to whole population screening is targeted screening of high-risk groups. Whether this would detect a clinically and publicly acceptable proportion of disease, and whether it would improve cost-effectiveness are unknown. The aim of this research is to estimate the clinical outcomes and cost-effectiveness of targeted AAA screening.

**Methods:**

Rather than conducting an expensive and time-consuming randomized trial to directly test targeted screening, we will undertake in-silico trials of targeted AAA screening. To determine success criteria for in-silico trials, the ethics and issues around the acceptability of targeted screening will first be explored through focus groups and interviews. A qualitative evidence synthesis to identify issues associated with targeted screening will be used to establish themes and topic guides. To perform the in-silico trials, individual men’s outcomes from the NAAASP (2013–2024, ≈ 2,500,000 men, ≈ 1% with AAA) will be linked to primary care data from the Clinical Practice Research Datalink (CPRD) (20% overlap of records). Risk factors for AAA will be identified by developing a risk prediction model and used as targeted screening criteria in in-silico trials, with diagnostic accuracy as the primary outcome. A discrete event simulation model will be adopted to extrapolate the trial findings beyond the observed period. We will estimate the clinical and cost-effectiveness of targeted screening compared with the current whole population screening strategy. Data linkage will be undertaken under Health Research Authority Confidentiality Advisory Group (Section 251) approval. Linked data will be effectively anonymised. Participants in the qualitative substudy will provide informed consent for participation.

**Discussion:**

We expect this project to have a direct and significant impact on NHS, UK and worldwide AAA screening policies. The study findings will be submitted for publication in peer-reviewed journals and presented at scientific meetings.

## Background

Abdominal aortic aneurysm (AAA) is a significant cause of mortality and morbidity. In England and Wales each year AAA rupture causes more than 3,000 deaths and over 6,000 patients undergo surgical AAA repair to prevent rupture [1,2]. The MASS trial of AAA screening, the key study on which current AAA screening practices are based, demonstrated a 52% reduction in AAA-related mortality in men screened for AAA [3] resulting in the introduction of screening for men aged 65 across the UK and elsewhere [4]. Further trials performed since the MASS trial have confirmed the long-term effectiveness of screening in reducing the risk of death from ruptured AAA and an additional benefit in terms of reducing all-cause mortality in men who attend screening [5].

The NHS AAA Screening Programme (NAAASP), which is the biggest provider of AAA screening in the UK, invites all men in the year of their 65^th^ birthday for a one-off ultrasound scan to screen for AAA. The majority of men screened for AAA do not have an aneurysm (approximately 1% in 2018 [6]); therefore, a significant proportion of the cost of screening is spent on screening those men who do not have the disease. Unnecessary screening of healthy individuals has both considerable financial implications and negative psychological effects [7].

AAA prevalence is the key determinant of screening cost-effectiveness. AAA prevalence in NAAASP is lower than that in the MASS trial. Screening men for AAA is cost-effective at current disease prevalence [8], however prevalence is reducing over time [9] (Table 1). As disease prevalence falls over time, screening will become less and less cost-effective. Ultimately this may result in de-commissioning of the programme and places those men with occult AAA at risk of death from AAA rupture.

**Table 1:** Reducing prevalence of AAA over time in the NHS AAA Screening programme between years 2013 and 2023.

Screening year:	2013/ 2014	2014/ 2015	2015/ 2016	2016/ 2017	2017/ 2018	2018/ 2019	2019/ 2020	2020/ 2021	2021/ 2022	2022/ 2023
**Men invited for screening:**	300,889	293,779	284,583	281,575	282,583	292,629	288,429	210,800	274,800	314,900
**Men attending for screening:**	235,409	233,426	227,543	223,371	222,887	237,416	222,177	164,002	217,092	254,439
**Attendance:**	78.24%	79.46%	79.96%	79.33%	78.87%	81.13%	77.03%	77.80%	79.00%	80.80%
**Men with AAA (>3.0 cm):**	2,941	2,773	2,549	2,387	2,232	2,309	2,033	1,542	1794	1,942
**AAA prevalence:**	**1.25%**	**1.19%**	**1.12%**	**1.07%**	**1.00%**	**0.97%**	**0.92%**	**0.94%**	**0.83%**	**0.76%**

The critical threshold for cost-effectiveness of AAA screening is a disease prevalence in the region of 0.35%−0.55% amongst those screened [5,10]. Whilst current prevalence is higher than this estimate at just under 1%, the falling AAA prevalence in NAAASP suggests that AAA screening may become unviable for the NHS within the next 10–15 years. In addition, studies highlighting the risks around AAA screening, such as overdiagnosis suggest that screening may be causing more harm than benefit [11,12], also contribute to the clinical argument against AAA screening. Therefore, it is important to investigate alternative screening strategies now, well before prevalence falls to a level where screening is ineffective on economic grounds alone.

Targeted AAA screening has the potential to improve cost-effectiveness and ensure the longevity of the AAA screening programme but needs to be tested. Any targeted screening strategy is likely to be based, at least in part, on AAA risk factors such as smoking. This approach has been utilized in the recent United States Preventative Services Taskforce (USPSTF) recommendation to only offer AAA screening to men with a history of smoking, stating that offering AAA screening to men who have not smoked is only of marginal net benefit and should not be routinely offered [13].

Targeted AAA screening is unlikely to detect the same proportion of AAAs as a whole population screening strategy. This means that some men with AAA who would have been offered screening in a whole population screening programme will not be offered screening and will be at risk of fatal AAA rupture. Socio-economic deprivation affects AAA screening uptake [14]. There is lower uptake among men living in deprived areas, precisely the areas in which AAA is more prevalent [15]. There is therefore the potential for stigmatisation, inequity and loss of justice with targeted screening. Therefore, the public acceptability of, and ethical issues raised by, targeted screening require exploration.

### Aims and objectives

This study aims to determine the clinical and cost-effectiveness of targeted AAA screening. Rather than conduct an expensive and long randomized trial, we propose to use a statistical modelling approach using real-world screening data to investigate targeted AAA screening. We will use the outcome and risk factor data to re-parameterise an established discrete event simulation statistical model of AAA screening to determine the clinical and cost-effectiveness of targeted AAA screening. Alongside this, we will also conduct a qualitative evidence synthesis review and focus group discussions to understand the ethical aspects and public acceptability of targeted screening. This will be used to set the success criteria for the quantitative analyses and frame our overall project results.

## Methods

### Study design

In this research to undertake a quantitative analysis of targeted AAA screening we will link individual patient screening outcome data from NAAASP to individual men’s primary care records available in the Clinical Practice Research Datalink (CPRD). This dataset will be used to determine the clinical outcomes of targeted AAA screening strategies had these been used. An established statistical model of AAA screening will be used and modified to compare the long-term harms, benefits, clinical effectiveness and cost effectiveness of targeted screening with the current population screening strategy.

This research will be conducted in four separate work packages:

Work package 1: Linkage of 2013–2024 NAAASP individual patient screening outcome data with primary care records from the CPRD and dataset cleaning/processing.

Work package 2: Identification/confirmation of AAA risk factors for screen-detected AAA in primary care records. Establishment of hypothetical targeted screening strategies and building a primary care risk prediction model for screen-detected AAA.

Work package 3: In-silico diagnostic accuracy trials of the hypothetical targeted screening strategies. Update an established discrete event simulation model of AAA screening. Use the outputs from the in-silico trials as new model parameters to estimate long-term changes in benefits, harms and cost-effectiveness of targeted screening vs whole population screening.

Work package 4: A qualitative study of the ethics and acceptability of targeted screening for AAA running alongside work packages 1–3. Consisting of a qualitative evidence synthesis and focus groups including men with men who benefited, or will benefit for the screening, and members of the public.

### Work package 1 (WP1)

**Fig 1:**
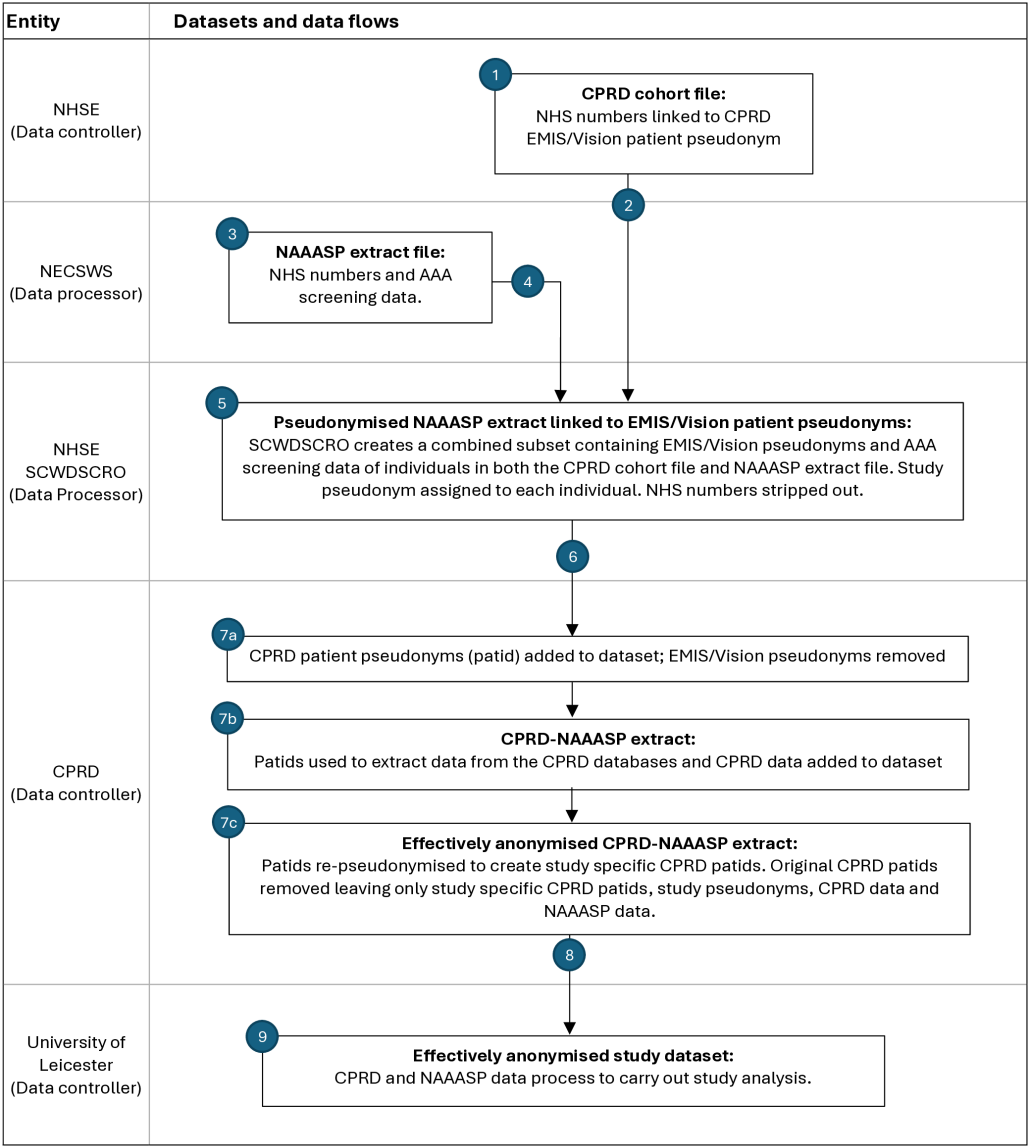
Data flow diagram for data linkage between NAAASP and CPRD. NHSE – NHS England, NECSWS – NEC software solutions, SCWDSCRO – South Central & West Data Services for Commissioners Regional Office, CPRD – Clinical Practice Research Datalink, EMIS/Vision – GP software from where data derives, CAG – health research authority confidentiality advisory group.

WP1 will involve the linkage of individual men’s data from the NAAASP with individual primary care records (Fig 1). The linked dataset will be prepared for analysis, including the conversion of coded primary care data into clinically meaningful research-ready data.

Data from the NAAASP will cover an eleven-year period from 2013 to 2024, thus we expect to have screening records for over 2,500,000 men invited for screening and over 25,000 men with AAAs. Primary care data will be obtained from the Clinical Practice Research Datalink (CPRD). CPRD is a database of anonymised primary care medical records covering over ~19 million patients from 1927 different UK GP practices [16–19]. Coding of clinical events in CPRD has been both technically and clinically validated [20–26]. We estimate that at least 20% of men invited for screening between 2013 and 2024 will be currently registered with GP practices contributing to CPRD.

We will undertake data linkage using the CPRD’s established pathway and patient level data-flow model for non-standard linkages. For this study both CPRD Aurum and CPRD GOLD datasets will be extracted.

The linkage will be conducted as follows (numbers in brackets refer to the numbers contained within Fig 1); (1) CPRD will instruct GP software suppliers, EMIS and Vision, to share NHS numbers and GP software (EMIS/Vision) patient pseudonyms for CPRD contributing practices with NHSE (the CPRD cohort file). (2) NHSE will share the CPRD cohort file with the South Central & West Data Services for Commissioners Regional Office (SCWDSCRO). This is an organisation within NHSE which will carry out linkage itself. (3) NAAASP via their software provider NEC Software Solutions will extract data from the NAAASP database according to a predefined data specification. (4) NAAASP will then share this extract file with SCWDSCRO. (5) SCWDSCRO will link the CPRD cohort file and NAAASP extract by NHS number, filtering and removing those men not within the CPRD cohort file. CPRD identifiers (the GP software patient pseudonyms) are added to the remaining NAAASP records. A new pseudonym is generated for each individual and the NHS numbers are stripped from the dataset. (6) This datafile is passed to CPRD. (7a) CPRD will use the GP software pseudonyms to add the CPRD patient pseudonyms to the dataset and then remove these GP software pseudonyms. (7b) CPRD patient pseudonyms are used to add the CPRD data which is added to the dataset. (7c) The whole dataset is then re-pseudonymised by creation of a new CPRD patient ID that is study specific and removal of all other pseudonym IDs except the study pseudonyms and newly generates study specific CPRD patient IDs. (8) This dataset will contain the CPRD and NAAASP linked data and is passed onto the University of Leicester. (9) University of Leicester perform study analysis.

### Work package 2 (WP2)

The aim of WP2 is to determine what the main risk factors for screen-detected AAA are, using the linked dataset generated in WP1 and thus develop hypothetical targeted screening strategies.

Known and putative AAA risk factors (smoking, hypertension, hyperlipidaemia, diabetes (protective factor)) will be tested for association with screen-detected AAA to determine their potential for use as sole criteria for targeted screening. Multivariable risk prediction modelling will be used to identify a set of clinical variables that can be used as criteria for targeted screening. Predictors for inclusion in the model will be selected based on mathematical/statistical performance and no predictors will be forced into the model based on prior assumptions.

If the final model is based on a small number of variables, adaptation for clinical and public use will also be considered. The target population for the model will be the same as the population in which it is developed, ensuring the relevance of the model to future clinical practice.

The primary performance criterion for the model will be achieving a predefined sensitivity threshold for detecting true positive cases in targeted AAA screening. This success criterion and/or the success threshold may be revised depending on the outputs from ethical study and focus groups. Sensitivity analyses to test variable selection strategies will be performed and the TRIPOD checklist will be used to present overall model results [27].

### Work package 3 (WP3)

Work package 3 consists of the following three tasks: Reviewing and updating an established discrete event simulation model (DES) of AAA screening, conducting in-silico trials of targeted vs population screening for AAA to determine the diagnostic accuracy of targeted screening and modelling the long-term effectiveness of targeted vs population screening using DES modelling.

In the ‘Screening Women for aortic ANeurysm’ (SWAN) project [10], a new DES model of AAA screening was developed to estimate the clinical and economic effectiveness of screening over a 30-year period [28]. The model provides outputs in terms of the number of clinical events, life-years gained with screening and incremental cost-effectiveness ratios (cost per quality-adjusted life-year gained) for a follow-up period of 30 years after screening. The model allows individual screening parameters to be varied and has been used to estimate the clinical and economic effects of varying surveillance intervals for men with small AAA in the NAAASP [28]. In this study, we will adopt the DES model to estimate the effectiveness and cost effectiveness of targeted AAA screening compared with whole population screening. We will populate the model using the data from in-silico trials.

The linked NAAASP-primary care dataset will be used to test the targeted AAA screening strategies developed in work package 2. This will conform to the following clinical trial structure:

(P)opulation: Men in the year of their 65th birthday (current AAA screening population)

(I)ntervention: Risk-factor targeted invitation for AAA screening

(C)omparison: Whole population screening

(O)utcome: Diagnostic effectiveness of targeted screening

In-silico trials will then be run using the full range of targeted screening strategies determined in work package 2. Significant risk factors (such as smoking) will be examined as criteria for screening, both in isolation and in combination. The primary outcome for these in-silico trials will be diagnostic effectiveness (sensitivity and specificity, positive and negative predicative values, likelihood ratio, diagnostic odds ratio and area under the receiver-operator characteristic curve). Secondary outcomes will be those required to re-parameterise the DES model (re-invitations, attendance, non-visualisation, AAA size distribution at screening, AAA prevalence at screening and proportion of AAAs detected). For each screening strategy, individuals will be categorized into invited and non-invited groups. In each of these groups, the actual screening outcomes will be known and using the true detection rate the overall proportional detection rate for each hypothesized screening strategy can be calculated.

The trial outputs from each targeted screening scenario examined will be compared for their effect on long-term clinical and cost-effectiveness. Absolute numbers of clinical events for the varying screening scenarios can be calculated using the DES model and used to compare clinical outcomes using the reference case scenario of whole-population invitation for screening as a comparator. Clinical results will be presented as changes to the number of clinical events, principally proportional AAA detection and AAA mortality but including the effect of quality-adjusted life years. Outcomes will be presented as the incremental cost per quality adjusted life gained and incremental net monetary benefit (INMB) between targeted screening and whole population screening. Sensitivity analyses will be conducted to explore the key assumptions used in the model; the effect of varying the methods for calculating smoking status, hypertension and hyperlipidaemia will be examined as well as additional sensitivity analyses for coding variations in any strong predictors of AAA identified in the risk prediction modelling exercise. Additional analyses relating to the management and delivery of both AAA screening and vascular surgical services will be considered on a case-by-case basis.

### Medical ethics work package 4

Alongside work packages 1–3, a qualitative study will be run to explore ethical issues and the public acceptability of targeted screening. This will assist us with the framing of results from the project and feed into the quantitative data analyses. The key question that this work package will address is what degree of underdiagnosis is acceptable in AAA screening programmes if costs need to be reduced to maintain viability for the NHS. We will establish what particular qualities of the screening programme that the public value, and whether there is potential for stigmatisation with targeted screening approaches. This study will be used to propose success criteria for the later quantitative work packages.

This study will take place in two stages, firstly a qualitative evidence synthesis review will be undertaken to identify ethical issues associated with targeted screening and establish themes and topic guides for the second stage of this research where focus group discussions will be used to explore the ethics and acceptability of targeted AAA screening. Focus group discussions may be supplemented with individual participant interviews either to follow-up with focus group participants or with additional participants who preferred not to attend focus group meetings.

Focus groups will be undertaken with four key group types; men who have already undergone AAA screening, men who are due to be invited to national AAA screening, men who did not attend screening but were invited to screening and members of the public (including young age groups and women). There will be at least two different focus groups run for each key group type. The authors expect difficulty in recruiting more than one focus group for men who did not attend screening and thus only one focus group may be performed for this group type.

Nearing study completion, our study results will be presented to and discussed with a patient and public involvement group (PPI) in order to obtain their views on the research findings. Together, the research team and the PPI group will craft a policy approach based on the findings with the aim of developing a set of public acceptability thresholds for the diagnostic accuracy of targeted screening to take forward the outputs from the project as a whole.

### Sample size calculation

Allowing for a minimum of 20% data availability in CPRD, linked data will be available for just over 500,000 men, with over 5,000 AAA cases. We expect our most basic model of targeted screening based on smoking status alone to have a sensitivity for AAA detection of between 45% and 85% [29,30]. Our sample size of 5,000 AAA cases has adequate power to estimate sensitivities of 70% and above with a marginal error of +/- 1.5% and sensitivities of 75% and above with a marginal error of +/- 1.25% (Hajian- Tilaki method [31], α = 0.05). To develop an overall risk stratification model using primary care data (work package 2), having 5,000 men with AAA (events), we have an adequate sample size to build and validate a model with over 20 predictor variables (based on a 4:1 training:testing split) [27,28]. Recognizing that events-per-variable (EPV) thresholds have limitations and can be misleading as a sole basis for determining sample size, we have used more contemporary method of sample size estimation based on the 4-step method of sample size prediction by Riley et al [32]. Accordingly, the study will have sufficient data for the development, internal validation, and testing of the proposed risk stratification model.

### Ethics approval and consent to participate

This study has Health Research Authority Confidentiality Advisory Group Section 251 approval to establish a legal and ethical basis for linking data. In addition, this study has Health Research Authority ethical approval via the West Midlands South Birmingham Research Ethics Committee, reference 18/WM/0140. The data will be kept anonymised after data-linkage. Qualitative substudy participants will provide written informed consent for participation. The consent process includes provision of participant information sheet with the opportunity to ask questions. Only adults aged 18years and over will be invited and included.

Regarding current progress at time of publication, the data linkage for this study is currently in progress and has not yet been completed. Recruitment for focus groups has been initiated on 25/05/2023 with predicted completion of recruitment 01/05/2026. Data collection and linkage is estimated to be completed 01/07/2025. The predicted study end date with final results for the study is 01/11/2026.

## Discussion

This will be the first virtual in-silico trial comparing targeted screening and population screening of AAA. The objective of the study is to assess the clinical and cost-effectiveness of targeted AAA screening, aiming to establish its viability as a solution to the declining cost-effectiveness of population-based AAA screening programs. Patient and public engagement has been central to the study from its inception and will remain integral throughout its implementation. Focus groups consisting of beneficiaries of screening and members of the public will help frame the success criteria and acceptability thresholds of the quantitative outputs, enhancing the relevance and applicability of the study’s findings.

This project’s strengths are founded on its mixed-method approach, utilization of a substantial real-world dataset (the largest linkage of national screening data to date), application of an established statistical model (DES model) combined with strong patient and public involvement. Multiple work packages provide a multimodal overview to address the research question.

Only an estimated 20% of the entire dataset will be available for linkage with primary care data, which itself has numerous inherent limitations (data incompleteness, coding variability, and selection bias). Despite this, the large sample size will have adequate sensitivity and power to develop, validate and test proposed risk stratification models. Multiple strategies may have to be employed to account for potential missing data. While multiple imputation remains one of the more widely used approaches, its appropriateness in prediction settings is debated. Multiple imputation generally assumes data is missing at random which is unlikely to be true in primary care records. Therefore, multiple imputation may introduce bias and limit generalisability. Sensitivity analyses will be essential to evaluate model performance across key subgroups and it will be important to explore if missingness itself carries a predictive value.

It is also possible that the predictive models generated in work package 1 do not maintain sufficient disease detection rates and that the best predictors for these models are obtained from primary care data recorded after the AAA screening appointments. Further subset (including temporal) and sensitivity analysis will be performed to account for this. The DES model, published in 2018, requires updating and contains multiple inherent assumptions. This will be updated in the study with up-to-date parameters reflecting the current modern natural history of AAA, screening compliance and costs.

Strategies for targeted AAA screening have previously been investigated in Australian and Danish screening study datasets [33,34]. Whilst these studies both concluded that disease detection rates for targeted screening were too low to be acceptable, these studies were based on a different age range of men than that invited in NAAASP, were performed at a time when disease prevalence was higher, and did not examine the use of primary care data as a method for cohort identification. Furthermore, no cost-effectiveness analyses were performed in either study and no other economic assessment of targeted AAA screening has been performed. No randomized controlled trials have been performed for targeted screening. Further research is needed to assess the feasibility, effectiveness, and cost-effectiveness of targeted AAA screening. This study aims to address these gaps.

We expect this project to have a direct and meaningful impact on NHS, UK and worldwide AAA screening policies. Our dissemination strategy will be to target those involved in screening policy decisions and the public via conventional media. The study findings will be submitted for publication in peer reviewed journals and presented at scientific meetings.
